# Comparison of the stated religious beliefs amongst UK intensive care physicians and the UK population

**DOI:** 10.1186/cc9937

**Published:** 2011-03-11

**Authors:** A Tillyard, DT Ashton-Cleary

**Affiliations:** 1 Derriford Hospital, Plymouth, UK

## Introduction

The UK is a multifaith culture and 77% of the population considered themselves to belong to a religious group in the 2001 UK Census [1]. Differing faiths have differing customs and views surrounding end-of-life decisions and care. Treatment withdrawal and withholding of life-sustaining care or CPR have been shown to be significantly influenced by both patient and physician religion [2]. We wanted to determine whether the population faith mix was reflected amongst UK intensive care physicians.

## Methods

We conducted an online survey amongst the members of the UK Intensive Care Society. We asked them to state whether they considered themselves to belong to a faith group.

## Results

A total of 550 questionnaires were returned; 182 (33.1%) were from intensive care consultants. These are compared with UK 2001 Census data. Over 50% abstained from the question (vs. 7.8% in the Census). A total 11.8% of respondents were atheists (vs. 15.05% in the Census). Members of the Catholic Church and Church of England formed 8.4% and 10.2% of respondents. These faiths are grouped together in the Census as Christians and formed 71.8% in that sample. A total 1.8% were Hindu (0.98% in the Census) and 1.5% were Muslim (vs. 2.78% in the Census). Those belonging to other faiths formed 14.5% amongst respondents and 1.59% in the Census.

## Conclusions

The proportionately smaller UK faith groups are represented to largely similar extents amongst physicians. A much larger proportion of our study sample abstained from the question than in the UK Census (51.8% vs. 7.8%). Our questionnaire was presented along with questions regarding decisions to exclude patients from the ICU; abstainers may have felt their religious beliefs were being unfairly judged as a source of bias in their other answers. The religious makeup of a group of physicians can clearly not be manipulated to match that of the population but consideration should be given to how this factor may influence treatment decisions. This is likely to be of particular relevance where physician and patient do not share the same faith.

## References

[B1] Office of National Statistics UK 2001 Census. Focus on Religion http://www.statistics.gov.uk/downloads/theme_compendia/for2004/FocusonReligion.pdf

[B2] Sprung CL JAMA 2003 290 790 797 10.1001/jama.290.6.790 12915432

